# Leptomeningeal Carcinomatosis Mimicking Reversible Cerebral Vasoconstriction Syndrome

**DOI:** 10.7759/cureus.22806

**Published:** 2022-03-03

**Authors:** Parneet Grewal, Julianne P Hall, Sumeet G Dua, Lauren Koffman, Rima M Dafer

**Affiliations:** 1 Department of Neurology, Medical University of South Carolina, Charleston, USA; 2 Department of Neurology, Rush University Medical Center, Chicago, USA; 3 Department of Radiology, Rush University Medical Center, Chicago, USA

**Keywords:** reversible cerebral vasoconstriction syndrome, leptomeningeal carcinomatosis, thunderclap headache, breast cancer metastasis, headache, headache disorders

## Abstract

Leptomeningeal carcinomatosis is the result of metastatic infiltration of the leptomeninges by malignant cells originating from an extra-meningeal primary tumor site. We describe a patient with active breast cancer who presented with thunderclap headaches (THs) and imaging showing multi-segment irregular arterial narrowing of intracranial vasculature.

A 58-year-old Caucasian woman with active stage IV estrogen receptor-positive breast adenocarcinoma and migraine presented with THs. Computed tomography and brain magnetic resonance imaging (MRI) without contrast were unremarkable. Over a period of one week, she had recurrent THs. Interval vessel imaging showed multi-segment irregular arterial narrowing. Treatment with verapamil was initiated for suspected reversible cerebral vasoconstriction syndrome (RCVS). She subsequently had two discrete episodes of confusion with aphasia and left upper extremity numbness. Repeat gadolinium-enhanced MRI showed nodular leptomeningeal enhancement. Lumbar puncture revealed malignant cells in the cerebrospinal fluid consistent with leptomeningeal carcinomatosis. She subsequently underwent whole brain radiation treatment and intrathecal chemotherapy and had no further episodes of TH.

Our case emphasizes the importance of considering leptomeningeal carcinomatosis in the differential diagnosis of THs and reversible cerebral vasculopathy, especially in patients with known underlying active cancer. The illustration also proves the importance of a complete work-up in patients with known malignancy in the setting of suspected RCVS.

## Introduction

Leptomeningeal metastasis (LM) is a result of metastatic infiltration of the leptomeninges by malignant cells originating from the extra-meningeal primary tumor site. It is most commonly a complication of breast adenocarcinoma, occurring in 12-35% of patients [1]. In this case report, we describe a patient with active breast cancer who presented with a clinical picture of recurrent thunderclap headaches (THs) with imaging showing multi-segment irregular arterial narrowing of intracranial vasculature in the setting of LM. Our case provides insight into the pathogenesis of this rare presentation, which has not been extensively studied in the past.

## Case presentation

A 58-year-old Caucasian woman presented with recurrent THs associated with nausea and vomiting. She had a history of stage IV breast adenocarcinoma post bilateral mastectomy and reconstruction surgery, with bone and liver metastases, on olaparib and enzalutamide. Other medical co-morbidities included non-Hodgkin's lymphoma in remission, migraine with visual auras, and hypertension. Computed tomography (CT) and brain magnetic resonance imaging (MRI) without contrast were unremarkable. No focal neurological deficits were noted on examination. She received a short course of high-dose oral dexamethasone, butalbital/acetaminophen/caffeine combination, and was started on gabapentin for presumptive diagnosis of an acute migraine attack. She was not started on any other acute migraine abortive medications.

Over one week, she had recurrent THs, and interval vessel imaging was completed. Magnetic resonance angiogram (MRA) showed multi-segment irregular arterial narrowing of the proximal and distal branches of the anterior and posterior arterial circulation. Based on her clinical course and imaging, treatment with verapamil was initiated for suspected reversible cerebral vasoconstriction syndrome (RCVS). Headaches transitioned from TH to a persistent headache and she developed visual auras, similar to her typical migraines, as well as binocular diplopia for one week. She subsequently had two discrete episodes of confusion with aphasia and left upper extremity numbness, prompting transfer to our tertiary care center.

**Figure 1:**
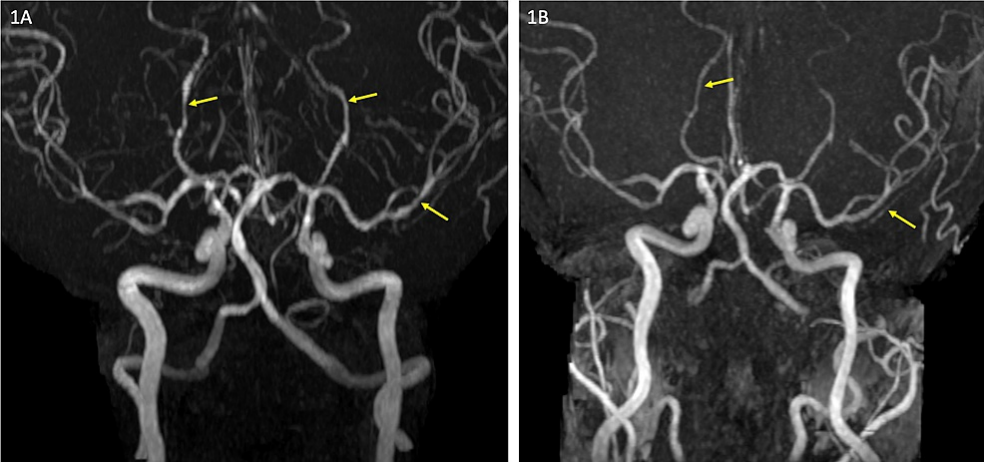
Magnetic resonance angiogram. (A) Maximum intensity reconstruction from time of flight magnetic resonance angiogram (MRA) showing segmental irregularity and narrowing involving the distal bilateral posterior cerebral arteries and left middle cerebral artery (arrows). (B) Follow-up MRA after six weeks shows complete resolution of the vascular abnormality.

Upon admission, repeat brain MRA showed similar arterial irregularity and narrowing, more prominent in the anterior than posterior intracranial vessels (Figure 1A). Brain MRI with contrast showed nodular leptomeningeal enhancement throughout the supratentorial and infratentorial compartments and a small area of restricted diffusion in the left inferior cerebellar hemisphere. MRI vessel wall imaging (black blood protocol) was normal. Lumbar puncture revealed an opening pressure of 200 mm of H2O, white blood cell count of 3, protein level of 24.9 mg/dL, a glucose level of 20 mg/dL, and negative cerebrospinal fluid (CSF) cultures. There were malignant cells in the CSF consistent with leptomeningeal carcinomatosis. This was confirmed with a repeat lumbar puncture. She was continued on oral verapamil therapy with plans for her to undergo whole brain radiation treatment and intrathecal chemotherapy after discharge. Complete resolution of headaches occurred within three weeks. Follow-up brain MRI and MRA at week six showed interval resolution of the arterial irregularity with a small area of gliosis in the left cerebellar hemisphere (Figure 1B).

## Discussion

The pathogenesis of LM is thought to be either hematogenous, due to the growth of the tumor along with nerve roots or arachnoid membrane, or as a direct spread from brain parenchyma [1]. The CSF cytological examination is the gold standard of diagnosis with 45-55% of patients having malignant cells detected in the CSF [1].

Several mechanisms, often combined, are implicated in the typical symptom-complex characteristic of LM, including hydrocephalus with elevated intracranial pressure, compression and invasion, ischemic injury, metabolic competition, and disruption of the blood-CSF barrier due to tumor angiogenesis [1]. The most common etiology of ischemic cerebral injury in LM is thought to be due to invasion, compression, or spasm of blood vessels along the brain convexity or in the Virchow-Robin spaces. This invasion of cells then interferes with the blood supply and oxygenation of neurons [2]. While vessel imaging is rarely performed in LM, focal arterial narrowing has been known to occur. A single-center experience of 90 patients with leptomeningeal metastases found an irregular arterial narrowing in four patients, suggesting either spasm of the pial vessels or infiltration of their walls by leptomeningeal disease [3].

Our patient presented with a recurrent TH, which is pathognomonic of RCVS, with typical reversibility of vasoconstrictive and ischemic changes on MRI and MRA along with the resolution of clinical symptoms [4]. The pathophysiology of RCVS has been postulated to be sympathetic overactivity, dysregulation of vascular tone, or blood-brain barrier (BBB) breakdown [5]. On review of current literature, anti-neoplastic drugs especially intrathecal chemotherapy regimens, are commonly implicated as the causality for RCVS. To our knowledge, anti-neoplastic agents that our patient was receiving (olaparib and enzalutamide) have not been associated with arterial changes [6] and she was not on any migraine abortive medications at the time of presentation, which can lead to RCVS. Migraine by itself also has a complex relationship with RCVS but the headache experienced by our patient was not typical of her migraine attack. Although not an established cause of RCVS, we hypothesize that the BBB breakdown due to the development of tumoral angiogenesis or invasion of tumor in the Virchow-Robin spaces in patients with LM could lead to segmental arterial narrowing and reversible vasculopathy that can mimic RCVS.

## Conclusions

Our case emphasizes the importance of having LM in the differential diagnosis of THs that are classically associated with other etiologies like RCVS. The illustration also proves the importance of a complete work-up, including contrast-enhanced MRI and CSF analysis in patients with known malignancy in the setting of suspected RCVS.
